# Synergistic Effect of Expressed miR-128 and Puma Protein on Targeted Induction of Tumor Cell Apoptosis

**DOI:** 10.15171/ijb.1429

**Published:** 2016-09

**Authors:** Shahryar Khoshtinat Nikkhoi, Ruhollah Dorostkar, Saeed Ranjbar, Hedieh Heydarzadeh, Mahdi Tat, Majdedin Ghalavand, Alireza Farasat, Mohammad Sadegh Hashemzadeh

**Affiliations:** ^1^Department of Medical Biotechnology, Faculty of Medical Sciences, Tarbiat Modares University, Tehran, Iran; ^2^Applied Virology Research Center, Baqiyatallah University of Medical Sciences, Tehran, Iran; ^3^Department of Microbiology, Azad University of Shahrehe-Qods, Tehran, Iran

**Keywords:** AAV, Adeno-Associated Virus, Gene therapy, Puma, Suicide gene

## Abstract

**Background:**

Puma is a highly robust pro-apoptotic protein. The protein becomes activated by p53 ensuing beyond-repair DNA damage. Downregulation of SIRT 1, by miR-128, elevates activated p53 that foment Puma indirectly.

**Objectives:**

In the present study, we used two-expression Adeno-Associated Virus (AAV) system for co-expression of miR-128 and Puma in order to evaluate apoptotic response; both in the tumor and normal cells, respectively.

**Materials and Methods:**

Three recombinant AAVs constructs were generated. The First rAAV bearing Puma under the control of hTERT (p-AAV), the second construct designed such that to carry miR-128 downstream of CMV (mi-AAV), and the last construct comprises of the both CMV-miR-128 and hTERT- Puma. Real-Time PCR and western blotting were used to evaluate expression levels of the transduced genes.

**Results:**

MTT assay and DAPI staining shown suicidal effect of each recombinant AAV vectors. p-AAV cytotoxicity was recorded for 62% of the tumor cells, while for normal cells it was only 20% cytotoxic. The second construct, mi-AAV, was not as potent and selective as p-AAV. This construct was shown to be 27% and 16% cytotoxic for BT-474 and HEK-293 cells, respectively. Co-expression of Puma and miR-128 (p-mi-AAV) was accomplished with a selective cytotoxicity toward BT-474. This construct was 85% toxic for tumor cells, although it was only 25% toxic for the normal cell line (HEK-293).

**Conclusions:**

In this study, we have shown that not only Puma is able to instigate apoptotic response but also its co-expression along with miR-128 could significantly enhance apoptosis in a synergistic manner.

## 1. Background

Adeno-associated virus (AAV) vectors are among the safest gene carriers. Firstly, these vectors elicit no immunogenic response, and secondly, they show insignificant amounts of unintended integration into the genome. The applications of AAV vectors in cancer therapy fall into several categories, such as delivery of the anti-angiogenic factors, suicide genes, as well as genes that are mediating genome repair. Among the drawbacks of AAV is its limited capacity for cloning. Virus production is tremendously reduced for genes with a size more than 5 kb. This disadvantage of AAV vectors has been resolved by a couple of advanced strategies such as using dual vectors (1), trans-splicing strategy (2,3), and overlapping vectors that exploit homologous recombination (4). Mitochondrion plays an essential role in the cellular respiration. This organelle is the major site for energy (ATP) generation. Besides, mitochondria are in the center of apoptosis through the intrinsic pathway (5). Subsequent to irreversible DNA damage, p53 is activated and induces apoptosis by permeabilization of the outer membrane of the mitochondria which eventuates in the release of cytochrome C into the cytoplasm where it functions as apoptotic protease activating factor 1 (APAF-1) to activate initiator caspase-9, an enzyme that subsequently cleaves and activates the executive caspases-7 (6).

The BCL-2 family of proteins directly regulates apoptosis. This family is composed of three members, anti-apoptosis (BH1-4), pro-apoptosis (BH1-3), and BH3 only proteins (7). BH3 only proteins heads the cells toward apoptosis by restraining anti-apoptotic modulators (e.g., BCL-2, BCL-xL and/or MCL^-1^) and activating pro-apoptosis proteins (*i*.*e*. BAK and/or BAX). Among BH3-only members, Bim and Bid inhibit anti-apoptotic proteins, while Puma, Bad, and Noxa activate pro-apoptotic proteins which lead cells toward apoptosis (8). Puma is one of the potent members, because it interacts with all pro-apoptotic protein, while, other members have more specific and narrower target range.

Loss of p53 is the main cause of cancer development, as in half of the cancers, somatic mutations in p53 could be detected. Genomic instability and high level of mutations are the hallmarks of all cancerous cells. This protein prevents cancer development by inducing cell cycle arrest (through p21 activation) and apoptosis following to the genomic mutation (6,9).

Puma (p53 upregulated modulator of apoptosis) undergoes transcriptional activation by many signals, among which p53 is considered as a prominent activator (9,10). Following to irreparable cellular damage, p53 becomes phosphorylated and activated by two kinases, ataxia telangiectasia-mutated (ATM) and ataxia telangiectasia and Rad3-related (ATR) (10,11). When activated, p53 activates Puma and Noxa, leading the damaged cell to apoptosis in order to prevent transformation (12). It is believed that Puma interacts and/or stimulates Bax and Bak through Bid and Bim. However, it has been suggested that Puma can also support apoptosis by direct activation of Bax. Besides to the p53, Puma is modulated by p53-independent stimuli such as toxins, deregulation of the oncogenic expression, bacterial and viral infection, lack of growth factors or cytokines, as well (13,14).

## 2. Objectives

In this study, we have evaluated the effects of Puma and miR-128 to instigate apoptosis. Three constructs were assembled and assessed for their effects on inducing apoptosis. The first construct, p-AAV, is an adeno-associated virus that carries Puma gene under the control of the hTERT promoter; a cancer-specific promoter that prevents undesired Puma expression. The second construct, the mi-AAV, expresses miR-128 constitutionally under CMV promoter. CMV is a strong promoter from cytomegaloviruses. The final killer construct, p-mi-AAV, is a hybrid of the both earlier constructs; a dual expression system that coexpresses both the Puma and miR-128 concurrently. In this recombinant AAV, apoptosis is induced following to the expression of the both miR-128 and Puma. miR- 128 represses SIRT 1 (Sirtuin 1) (15). SIRT 1 is a deacetylase that removes acetyl group from the p53 promoter region and downregulates its expression (16). By inhibiting SIRT 1, p53 becomes more abundant for activating Puma in order to elicit the apoptotic response (17,18).

## 3. Materials and Methods

### 3.1. Cell Culture

BT-474 and human embryonic kidney (HEK) 293T cells were grown in Dulbecco’s modified Eagle’s medium (DMEM, Sigma-Aldrich) supplemented with 10% FBS, 100 units.mL^-1^ penicillin, 100 mg.mL^-1^ streptomycin, and incubated in 5% CO_2_ at 37ºC. During the passages cells were rinsed twice with 3 mL PBS buffer at room temperature. Cells were detached by 0.25% trypsin solution at 37ºC for 2-3 min and then stopped with 4 mL of DMEM culture medium (10% FBS).

### 3.2. Sub-Cloning of miR-128 into rAAV

miR-128 was synthesized and received in PUc18 vector (Pishgam Company, Iran). The miR-128 construct was double digested with the *Eco RI* and *NcoI* and then ligated in to the same site on AAV vector to generate mi-AAV. As a result, the *miR-128* gene was replaced cap and rep in rAAV vector downstream of CMV promoter.

### 3.3. Cloning of Puma into rAAV

*Puma* cDNA was synthesized and amplified using RNA extracted from HEK 293 cells. RNA was isolated from HEK 293 cells applying Qiagen RNeasy Mini Kit and cDNA was synthesized. Amplification was done through the application of the following primers: 5´-accggatccGCGGCGCGAGCC-3´ and 5´-actgcggccg cTGTTCCAATCTGA-3´ (the nucleotides that are shown with the lower case letters stand for restriction site for *Bam HI* and *No tI*, respectively). Following to the cDNA synthesis and amplification, PCR products were double-digested with the *BamHI*-*NotI*, and then were sub-cloned into pUC19. *Puma* encoding gene was fused to the downstream of the hTERT promoter by splicing by overlap extension PCR (SOE PCR) method. Puma and hTERT (that has already been subcloned in to p CDNA 3.1) were amplified by polymerase chain reaction. The amplified fragments were ligated together applying another round of PCR. The fused hTERT-Puma fragment was digested with the *SalI*-*NotI* and subsequently was ligated into the pUC- 19. The fused hTERT-Puma was further subcloned into both AAV and mi-AAV to generate p-AAV and p-mi-AAV respectively.

### 3.4. Production of Recombinant AAV Vectors

Approximately a week and a half before viral production, HEK-293 cells were grown in the high-glucose DMEM medium containing 10% FBS and 100 μg.mL^-1^ penicillin G-streptomycin at 37ºC supplemented with 5% CO_2_ incubator. Endotoxin-free plasmid (Thermo scientific) of the rAAV (p-AAV, mi- AAV, p-mi-AAV), and the adenovirus helper plasmid, pFΔ6, which provides the cloned vector with the adenoviral helper, were prepared. For a 15×150 mm plate preparation, 187.5 μg of the cis plasmid plus 562.5 μg of each helper plasmids were utilized. The three plasmids were transduced at the molar ration of 1:3:3 into the HEK-293 cells (seeded at a density of 1.3×105 cells.cm^-1^, 3 days before transfection) by means of calcium phosphate method.

Briefly, 1 to 2 h before transfection, each 10 cm diameter plate of the human 293 cells (at 50% of the confluence) was fed with 10 mL of the fresh DMEM containing 10% fetal bovine serum without antibiotics. A total of 25 μg of the plasmid DNA, both the helper plasmid and the plasmid harboring the gene of interest was dissolved in 1 mL of 0.25 M CaCl_2_ and then rapidly mixed with 1 mL of the HEPES-buffered saline (50 mM HEPES, 280 mM NaCl, 1.5 mM Na_2_HPO_4_ [pH 7.12]) and was added to the cells. About 8 to 12 h after transfection, the medium was exchanged with the fresh DMEM containing 10% fetal bovine serum and antibiotics. Three days post-transfection, the transduced HEK-293 were collected and lysed with repeated freeze/thaw cycles. The cell lysate was clarified using low speed centrifugation at room temperature and the rAAV particles were purified by the affinity chromatography using AVB Sepharose High Performance (GE Healthcare). The trapped rAAV particles were eluted from the affinity column with 65 mM glycine buffer (pH 2.7). The purified rAAV particles were concentrated to a volume of 500 μL and were dialyzed against 2x PBS/0.001% pluronic acid exploiting Amicon 100 k MWCO concentrators (Amicon, TX).

### 3.5. rAAV Characterization by the Real-Time PCR

The viral titer of the vector preparations, the amount of viral particles that include a single-stranded DNA, was estimated by standard quantitative realtime. In brief, the purified rAAV particles were treated with the DNAseI (15min at 25ºC) before viral DNA purification. Proteinase K was applied to digest viral capsid (60 min at 65ºC). Viral genome was then amplified with the rAAV specific primers WPREfor (ggc tgt tgg gca ctg aca at) and WPRErev (ccg aag gga cgt agc aga ag) using 2×SYBR Green master mix (Applied Biosystems) operating real time PCR instrument (Corbett RG 6000: 4 min at 95ºC ensued by 45 cycles of 10 sec at 95ºC, 15 sec at 60ºC, 2 min at 72ºC). A standard curve of the diluted plasmid equivalent to 3.9×10^9^ to 1×10^11^ vg was utilized to calculate virus titers of rAAV preparations. The primers for amplification of the control glyceraldehyde-3-phosphate dehydrogenase (GAPDH) were: the forward 5´-ctcagacaccatggggaaggtga- 3´ and the reverse 5´- atgatcttgaggctgttgtcata- 3´.

### 3.6. Transduction Confirmation

#### 3.6.1. Quantitative Estimation of the Puma Expression Using Real-Time PCR

Transducted BT-474 cells were grown in DMEM containing 10% fetal bovine serum. Cells were detached from the plate and washed twice in PBS. Then cells were mixed with the RTL (Qiagen RNeasy lysis buffer) lysis buffer. RNA extractions were carried out according to Qiagen R Neasy Mini Kit. Quantitative PCR was performed using miR-128 (for p-mi-AAV and mi-AAV) and Puma (for p-mi-AAV and p-AAV) exploiting specific primers and Syber Green master mix. GAPDH was amplified as a control using primers mentioned above.

#### 3.6.2. Western Blot Analysis

Cells were detached from the dishes by scraping and were harvested by centrifugation at 1200 rpm for 4 min, then were washed twice with the PBS, and resuspended in the NP-40 lysis buffer supplemented with the 1 mM PMSF (phenyl methyl sulfonyl fluoride) and 10 mM HEPES at pH 7.4. The supernatant was separated by centrifugation using 12000 ×*g* for 10 min, collected and protein concentration was determined using Bradford reagent according to Bradford’s method (19). Sixty microgram of the proteins was resolved by 10% sodium dodecyl sulfate-polyacrylamide gel electrophoresis (SDS-PAGE) (20) and then transferred onto nitrocellulose membrane via wet transfer method for 3 h on ice (21). The membrane was soaked in 7% skim milk 2 h for blocking. Target proteins were probed with the mouse anti-His-tag specific primary antibody and then with the HRP-conjugated goat anti-mouse IgG secondary antibody. The desired protein band was detected after chemiluminescence reagent was used to detect secondary probes.

### 3.7. Cell Viability; MTT Assay

The MTT colorimetric assay is based on the reduction of MTT reagent MTT (3-(4, 5-Dimethylthiazol-2- yl)-2,5-diphenyltetrazolium bromide) to a formazan salt. Viable cells are able to reduce MTT reagent, while, cells that have undergone apoptosis cannot.Three MTT assays were done five days after transduction. The MTT assays were done in triplicates. For each assay, cells were seeded into a 96-well plate at a density of 3×103 cells per well. Then cells were incubated in 500 μg.mL^-1^ MTT (Invitrogen) reagent for four hours. After formazan crystals formation, SDS/HCl was added in order to dissolve formazan and the absorbance was measured at 570 nm using a spectrophotometer.

### 3.8. DAPI Staining

Transducted cells were harvested by centrifugation at 12000 rpm for 3 min and then were fixed in a solution containing 3.7% formaldehyde, 0.5% Nonidet P- 40, and 10 μg.mL^-1^ 4´,6-diamidino-2-phenylindole (DAPI) in PBS (final concentration of each reagent). Apoptosis was evaluated by means of microscopic visualization of the condensed chromatin and micronucleation.

## 4. Results

### 4.1. Puma cDNA Cloning into AAV

**Figure 1:**
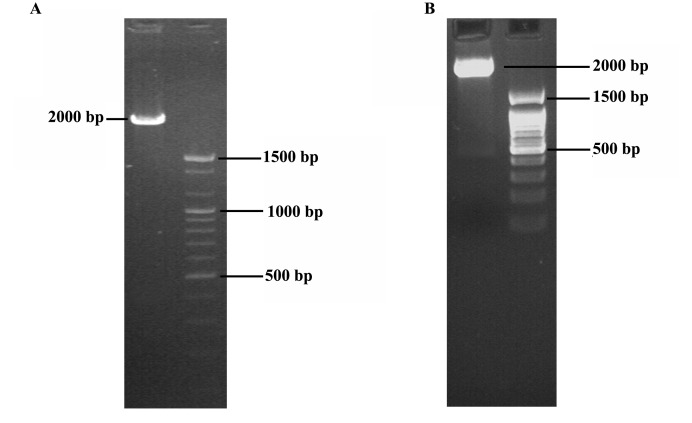
A: Puma mRNA was extracted and cDNA was synthesized and amplification was carried out using Puma specific primers. B: hTERT promoter (200 bp) and amplified Puma were ligated together applying SOE PCR

Puma RNA was extracted and cDNA was synthesized as described above. The amplified Puma cDNAwas gel extracted and cloned into AAV vector containing hTERT promoter (Figure 1).

### 4.2. Real-Time Polymerase Chain Reaction, Puma and miR-128 Expression

**Figure 2:**
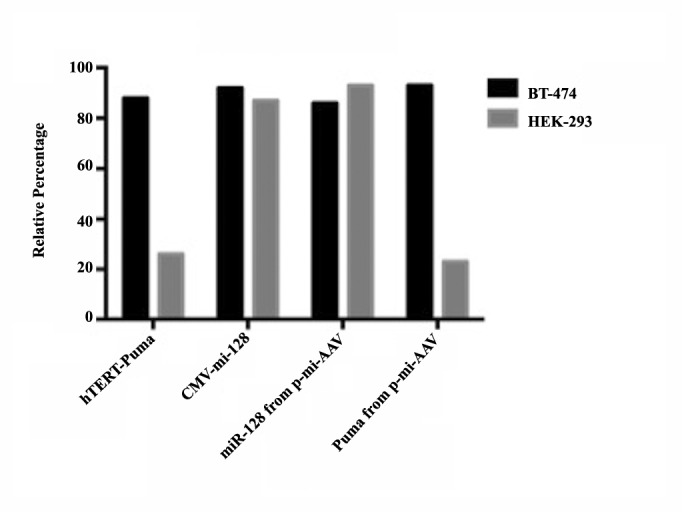
Real-Time PCR quantitatively evaluated the expression of Puma and miR-128 for the three viral particles. Puma is selectively expressed in the tumor cells, at least, three times higher than the normal cells, although miR-128 is roughly expressed at the same level in both cancerous and normal cells, respectively

Real-time PCR was used for quantitative assessment of the Puma and miR-128 expression. After RNA extraction from both the tumor and the normal trans duced cell lines, Real-time PCR was carried out. A high level of miR-128 expression was found in both cell lines because of CMV promoter which is a strong promoter derived from cytomegalovirus. Genes under CMV control are constitutionally expressed. Our results showed that Puma is selectively expressed in the tumor cell line. The relative expression of the Puma was 88% in the BT-474 tumor cell line. In contrast, its expression was found to be 26% in the normal cell line, HEK-293; an amount of expression about four times less (Figure 2).

### 4.3. Puma Expression Detection, Western Blotting

**Figure 3:**
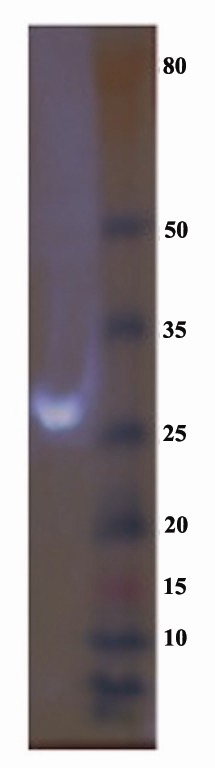
Puma western blotting. The transducted Puma expression was corroborated by western blotting. Anti-his tag monoclonal antibody probed 6 x his tag at the C-terminal of Puma. The band around 27 kDa shows Puma expression in BT-474 cell lines

In order to confirm Puma expression in BT-474 cells, Puma was probed with anti-His tag antibody. His tag was incorporated into C-terminal of Puma because we needed to distinguish between the cellular Puma protein and the Puma that was transduced using AAV. The band around 27 kDa was a proof for Puma expression (Figure 3).

### 4.4. Selective Apoptosis in Tumor Cells

**Figure 4:**
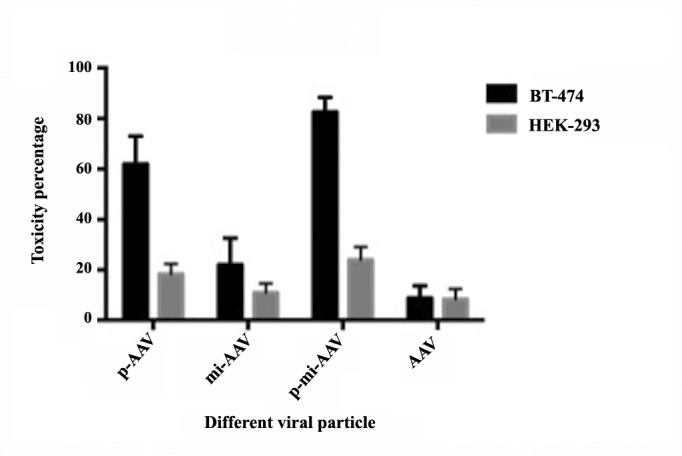
MTT assay was applied for evaluating the cytotoxic effects of the viral particle. The colorimetric cytotoxicity assay was done 5 days after transduction. Although mi-AAV did not show a significant selective toxicity, p-AAV, and p-mi-AAV have shown a significant difference in the cytotoxicity effects between cancerous cell line (BT-474) and the normal cell line (HEK-293), respectively

Ensuing transduction by the three viral particles (p-AAV, mi-AAV, and p-mi-AAV), MTT assays verified the highly selective apoptosis in tumor cell line (BT-474). MTT assay was carried out five days after transduction. As it is illustrated in (Figure 4), all the three viral particles have selectively commenced apoptotic responses in BT-474. The measured cytotoxicity of the p-AAV on BT-474 cells was 62%. This amount is 42% higher than the cytotoxicity observed in HEK-293 cells (20%). This difference in toxicity between these two cell lines is because Puma expression is directed by hTERT promoter; hence, this killer gene transcriptionally targets tumor cells. The toxicity effect of mi- AAV is not as much as p-AAV. The AAV equipped with miR-128 was toxic for 27% of the BT-474 cells and 16% of the normal cell line (HEK-293). The third viral particle, p-mi-AAV, showed a magnificent targeted cytotoxicity effect on the cancerous cell lines. These two-expression-AAV particles have demonstrated 82% of the cytotoxicity in BT-474 while 25% in the normal cell line. The promising result was acquired, as there was a big difference in the cytotoxicity between p-mi-AAV and p-AAV, but a negligible increase in the toxicity toward the normal cell.

### 4.5. Apoptosis Induction by p-AAV in Combination with miR-128

**Figure 5:**
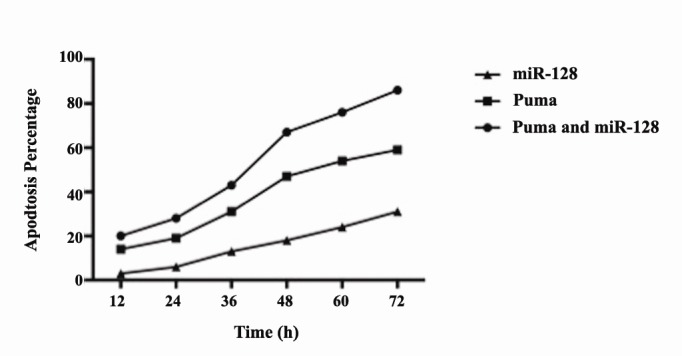
DAPI staining and estimation of apoptosis as measured by calculating the percentage of the apoptotic cells following to the transduction. Cells transduced with the p-AAV show 59% of apoptosis after 72 h. In contrast, only 31% of the cells underwent apoptosis ensuing miR-128 transduction. Transduction by p-mi-AAV showed higher apoptosis (86% of apoptosis correspondingly)

As illustrated in (Figure 5), p-mi-AAV (200 MOI) induces apoptosis in BT-474 cells greater than what does p-AAV (200 MOI) does. We also counted the apoptotic cells by DAPI staining after BT-474 cells were infected with 200 MOI alone and in combination with miR-128. The estimated apoptotic percentage that resulted from p-AAV and miR-129 were 59% and 31% respectively, 72 hours post-transduction. When a combination of the both genes was used the apoptotic percentages was increased by 86%. These results indicate that miR-128 infection facilitates apoptosis induced by Puma. The potent inhibition of the cell growth caused by the combination of Puma and miR-128 may result from more substantial apoptosis induction.

## 5. Discussion

Puma is one of the most potent BH3-only pro-apoptotic proteins that drive cells to the death destination. There are many Puma modulators, among which the most well distinguished: p53. Activated Puma inhibits anti-apoptotic proteins to accelerate MOMP (mitochondrial outer membrane permeabilization) that results in the cytochrome C and Smac/ DIABLO relocation into the cytoplasm, which triggers apoptotic response (12). Puma subjects to the activation following to the stress signals such as single- or doublestrand DNA break, γ radiation and topoisomerase inhibition, which occur when chemotherapeutic agents (e.g. doxorubicin) are used (14). Cancerous cells are resistant to the apoptosis following to the damages onto genomic DNA. Inducing apoptosis by gene therapy alongside with chemotherapy can enhance the efficacy of the therapy. Puma deficiency has been reported in many gliomas, neuroblastomas, certain types of B-cell lymphomas, as well as head and neck cancers. Puma deficiency is either due to breakage of the large arm of chromosome 19 (region encoding Puma) or its promoter methylation. Besides to these reasons, more than 50% of the tumors show a lack of p53: the Puma modulator. Taken together, we assumed that inducing expression of Puma under the control of tumor specific promoter (i.e. hTERT) alongside with the miR-128 which downregulates Sirtuin 1 (p53 promoter deacetylase), significantly enhances apoptosis in the cancerous cells.

Haijuan Wang *et al* (2006) have shown that transduced Puma, through p53, has largely enhanced apoptosis in esophageal cancer. This research team have used four cell lines with different p53 status and have shown that Puma could be a potent killer gene and inducer of apoptosis irrelevant to the p53. They also illustrated that Ad-Puma trigger apoptosis rather stronger than Ad-p53 in p53 deficient cell lines (8).

Jian Yu *et al*. (2006) experienced the similar results with a minor difference. They trans duced Puma using adenovirus to the lung tumor cells. They showed that induction of apoptosis following to the chemotherapy could be due to p53 dysfunction. After treatment with the chemotherapeutic agent, they have trans duced Puma into cancer cells. Results that they have obtained showed a higher rate of apoptosis following to the treatment with the chemotherapeutic agent. They concluded that over expression of Puma sensitizes cancerous cells to the chemotherapy (14).

In 2011, Leibowitz *et al* have designed an interesting study regarding Puma, p21, and p53. They found that Puma has a vital and important role in the induction of apoptosis following to the radiotherapy. They used mice with knocked out p53 and p21. These mice were divided into two groups: one group with a fully functioning Puma (+/+) and the other group with knockout Puma (-/-). After radiotherapy with 15 Gy, Puma (+/+) have survived for a longer time (10.5 days). Besides, mice with heterozygote Puma (+/-) survived longer than mice with completely knocked out Puma (-/-).

In this study, we scrutinized the potential of the Puma for inducing apoptosis with the expression of miR-128. miR-128 represses Sirtuin 1 mRNA translation. Sirtuin 1 is a histone deacetylase that removes the acetyl group from the p53 promoter, which leads to p53 downregulation (16).

MiR-128 induces apoptosis in BT-474 cells more than HEK-293 cells. Although miR-128 expression was under the control of CMV (constitutive expression), BT-474 cells underwent into apoptotic response more than the normal cells, because tumor cells proliferate and replicate much higher than normal cells. So, it is quite clear that rapid proliferation lead to more DNA damage and higher rate of apoptotic response than HEK 293T, which is a normal cell line with normal mutation rate (1 in 1010 bases).

As could be predicted, expression of Puma has elevated apoptosis in the tumor cells (BT-474) more than HEK 293T normal cells. Puma was under the control of hTERT, a promoter that is active exclusively in the transformed cell lines (22-25). Hence, Puma was expressed higher in BT-474. These findings indicate that we trans criptionally targeted Puma to prevent undesired damage to the normal cells. Selectivity, or therapeutic index, is crucial for any therapeutic agent. In the last group, co-expression of Puma and miR-128 has provoked apoptosis significantly higher than Puma and miR-128 alone. Furthermore, a trivial induction of apoptosis was recorded following to the transduction of p-mi-AAV into HEK-293 cells. The higher miR-128, the lower Sertuin 1 expression, which results in higher p53 expression (18). The higher the level of p53, the more activation of Puma would happen, and thus, an enhanced Puma-driven apoptosis could result. In the other words, not only Puma was successful to induce programmed cell death (PCD) in p53-independent approach but also through a higher expression of the p53, Puma was also able to induce apoptosis in a p53-dependent manner. Hence, Puma is using both pathways a higher percentage of apoptosis is expected rather than Puma which uses p53-independent route only.

## Acknowledgments

This study was supported by a fund from the Applied Virology Research Center, Baqiyatallah University of Medical Sciences (BMSU).
